# Acousto-optic stimuli to promote coherent 40-Hz frequency entrainment effect

**DOI:** 10.1055/s-0043-1777008

**Published:** 2023-11-30

**Authors:** Xue Han, Lei Wang, Shuo Yang

**Affiliations:** 1 Hebei University of Technology, School of Life Science and Health Engineering, Tianjin, China.; 2 Hebei University of Technology, State Key Laboratory of Reliability and Intelligence of Electrical Equipment, Tianjin, China.

**Keywords:** Alzheimer Disease, Electroencephalography, Memory, Short-Term, Doença de Alzheimer, Eletroencefalografia, Memória de Curto Prazo

## Abstract

**Background** Research has shown that a fundamental frequency of 40 Hz in continuous neural oscillation is indicative of normal brain activity; in Alzheimer disease (AD) patients, these oscillations either disappear or are significantly interrupted. Research has also indicated that the degenerative impacts of AD in mice were mitigated by the synchronization of 40-Hz acousto-optic stimulation (AOS).

**Objective** To examine the impact of employing a 40-Hz AOS intervention on the induction of a substantial 40-Hz frequency entrainment and improvement in working memory performance among a sample of young individuals in good health. We conduct an analysis of event-related potentials (ERPs) derived from electroencephalogram (EEG) data following the presentation of AOS.

**Methods** We recruited 20 healthy volunteers (median age: 25 years; 8 female subjects). Following the administration of various stimuli, including no stimuli, 40-Hz AOS, pink noise, and 40Hz acoustic stimuli (AS), the participants were required to complete a working memory task. A total of 62 electrodes were used to record EEG data, which was subsequently analyzed to investigate the impact of AOS on the activity of working memory. We also aimed to determine if AOS lead to a more pronounced 40-Hz frequency entrainment.

**Results** Following the administration of AOS, a notable enhancement in the 40-Hz power of pertinent cerebral areas was observed, accompanied by a substantial improvement in the performance of the subjects on working memory tests subsequent to the stimulation.

**Conclusion** The findings unequivocally establish the efficacy of using AOS to enhance the 40-Hz power and working memory.

## Resumo

**Antecedentes** A pesquisa mostrou que uma frequência fundamental de 40 Hz em oscilação neural contínua é indicativa de atividade cerebral normal. Em pacientes com doença de Alzheimer (DA), essas oscilações desaparecem ou são significativamente interrompidas. A pesquisa também indicou que os impactos degenerativos da DA em camundongos foram mitigados pela sincronização da estimulação acústico-óptica (EAO) de 40 Hz.

**Objetivo** Examinar o impacto do emprego de uma intervenção EAO de 40 Hz na indução de um arrastamento substancial de frequência de 40 Hz e na melhoria do desempenho da memória de trabalho entre uma amostra de jovens com boa saúde. Conduzimos uma análise de potenciais relacionados a eventos (PREs) derivados de dados de eletroencefalograma (EEG) após a apresentação de EAO.

**Métodos** Recrutamos 20 voluntários saudáveis (idade média: 25 anos; 8 mulheres). Após a administração de vários estímulos, incluindo nenhum estímulo, EAO de 40 Hz, ruído rosa e estímulos acústicos (EA) de 40 Hz, os participantes foram obrigados a completar uma tarefa de memória de trabalho. Um total de 62 eletrodos foram utilizados para registrar dados de EEG, que foram posteriormente analisados. para investigar o impacto do AOS na atividade da memória de trabalho. Também pretendemos determinar se o AOS leva a um arrastamento de frequência de 40 Hz mais pronunciado.

**Resultados** Após a administração de AOS, foi observado um aumento notável na potência de 40 Hz de áreas cerebrais pertinentes, acompanhado por uma melhoria substancial no desempenho dos sujeitos em testes de memória de trabalho subsequentes à estimulação.

Conclusão Os resultados estabelecem inequivocamente a eficácia do uso do AOS para melhorar a potência de 40 Hz e a memória de trabalho.

## INTRODUCTION

Based on the findings of the “World Alzheimer Report 2018,” a new case of dementia occurs approximately every 3 seconds on a global scale. The global population of individuals diagnosed with dementia is estimated to be of approximately 50 million, a figure that has been projected to increase to 150 million by 2050, with 60% to 70% of this population composed of AD patients.

Furthermore, along with the primary clinical characteristics of AD there is also a reported disruption in the oscillation of the neural network. 1 Research 2 has indicated that there are alterations in the gamma band (30 Hz to 80 Hz) oscillations associated with cognitive processes such as attention and memory in individuals diagnosed with Ad. Similar changes have also been observed in mouse models of the disease. 3 4

While pharmaceutical interventions have demonstrated the ability to provide temporary relief from the symptoms associated with AD, it is important to note that a cure for the condition is currently unavailable. 5 6 As research continues to progress and broaden, there is a growing fascination among individuals with non-pharmacological treatments, particularly those that involve the modulation of brain oscillations. 7 8 Research revealed a synchronization between the internal neural oscillation and external inputs, such as light, sound, and transcranial electrical stimulation. 9 10 External rhythmic stimulation has the potential of entraining the frequency and timing of the oscillation. 11 Research 12 has shown that a fundamental frequency of 40 Hz in continuous neural oscillation is indicative of normal brain activity; this frequency has been associated with an involvement in attentional processes and the functioning of memory. 13 The therapeutic approach of nerve oscillation control is promising due to its potential benefits to individuals with AD and other affected populations. 14

Motivated by the findings made by Chan et al., 15 Adaikkan and Tsai, 16 and He et al., 17 the present work investigated the application of non-invasive acousto-optic stimulation (AOS) to improve the synchronization of brain oscillations at a frequency of 40 Hz. Research 18 19 has indicated that the gamma band has a preference for memory retrieval in familiar situations, and the middle gamma band is activated to regulate the process of recall. Jones et al. 20 reported that, when patients are subjected to sonic stimulation at a frequency of 40 Hz, a broader spectrum of gamma brain oscillations is triggered in comparison to treatment at other frequencies within the gamma band. The administration of acoustic stimulation has been observed 17 to decrease the expression of a relatively ineffective activator of tumor necrosis factor-related apoptosis; this modulation of gene expression has been found to have an impact on neural activity and subsequently enhance cognitive performance in AD patients. The use of AOS results in the induction of more synchronized gamma oscillations, which have a more extensive and profound impact on various brain regions compared to the isolated use of acoustic or optical stimulation. 21 In an electroencephalography (EEG) investigation, Fatemi et al. 22 observed that the application of 40 Hz AOS resulted in a notable improvement in the power spectral density of neural oscillations in the frontal and occipital lobes, and this stimulation technique was able to induce gamma-frequency entrainment in deeper brain regions, as well as an increase in theta-gamma phase-amplitude coupling. In a functional magnetic resonance imaging (fMRI) investigation, the authors observed that acoustic stimulation at a frequency of 40 Hz resulted in a delay in brain atrophy. Additionally, this stimulation was found to enhance the functional connectivity between the entire brain and the medial visual network, as well as between the posterior cingulate cortex and the precuneus. The aforementioned findings collectively demonstrate that a frequency of 40 Hz is the optimal choice to decelerate or mitigate neurodegenerative processes, and to improve cognitive function through the induction of gamma oscillations.

## METHODS

### Participants

For the present study, we recruited 20 (8 female and 12 male) subjects with a mean age 25 years. Prior to the commencement of the study, all participants underwent a basic oral assessment to ensure the absence of achromatopsia, colorblindness, and any hearing impairments.

### Stimuli

In order to produce a 40-Hz AOS within the gamma frequency range, we developed an auditory signal using the Python programming language; we employed a modulation wave of 40 Hz, which was superimposed on a carrier wave of 250 Hz to serve as the acoustic stimulus. The optic stimuli used was a video in which black and white screens rapidly alternate at a frequency of 40 Hz. We used an image cropping software to seamlessly combine the two stimuli into an AOS.

### Visuospatial working memory tasks

**Figure 1:**
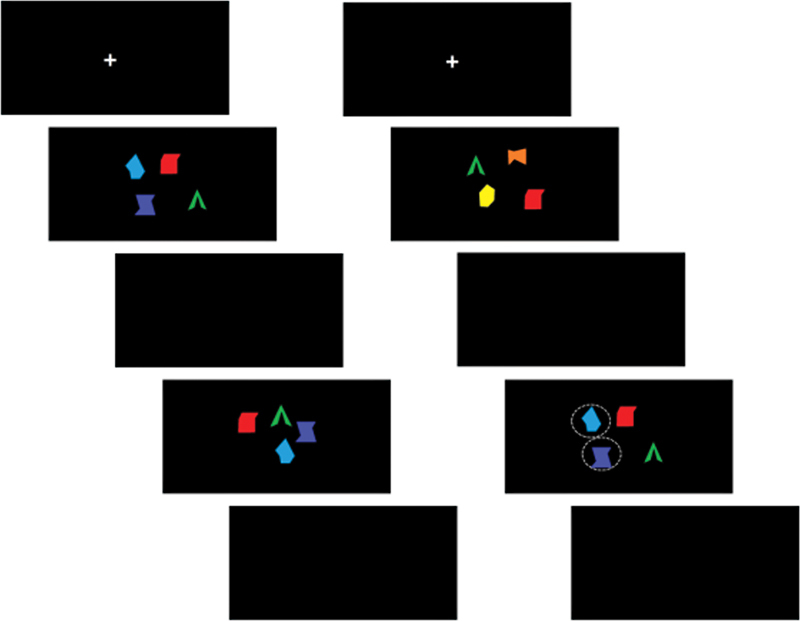
The left is a match, the right is a mismatch; a response was required in the fourth picture.

To evaluate the potential impact on working memory after the stimulation, the participants were subjected to a sequence of working memory performance tests that lasted either 5 or 8 minutes, with a visual delay matched accordingly. Throughout the entirety of the experiment, the duration of picture presentation in each encoding phase was of 1,500 ms, the retention period lasted for 1,500 ms, and the extraction period spanned 2,000 ms. The time interval between each set of photo test pairs was of 3,000 ms. In all experimental trials there was an equal distribution of target stimuli (50%) and non-target stimuli (50%). The participants were instructed to hit the left arrow key when a match was identified, and the right arrow key when a mismatch was detected. Figure 1 displays examples of the delayed matching-to-sample tasks used in the experiment.

### Procedure

The participants were instructed to engage in activities one day before the main experiment, and they were provided detailed information regarding the experimental procedures. It was imperative to mitigate the potential influence of the subjects' learning state on experimental outcomes by ensuring that the experiment commences only after the individuals have transitioned out of this phase.

The EEG signal was susceptible to contamination from noise and the formation of different artifacts, including electromyography artifacts, mains interference, electrocardiogram (ECG) artifacts, and ophthalmic artifacts, due to its high unpredictability and low amplitude. Consequently, the participants were instructed to refrain from performing bodily movements and excessive blinking while undergoing the EEG recording to minimize any interference with the integrity of the EEG data.

**Figure 2:**
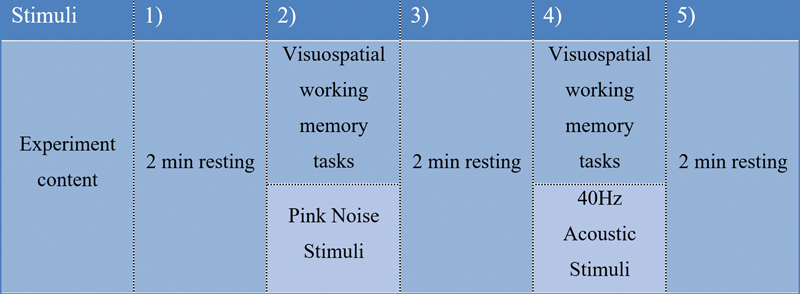
The experimental administration of acoustic stimuli.

The 40-Hz acoustic stimuli experimental session was divided into 5 segments: performance of the first resting EEG (duration: 2 m); working memory task with pink noise stimulation in the control experiment (duration: 5 m); performance of the she second resting EEG (duration: 2 m); working memory task with 40-Hz acoustic stimuli (duration: 5 m); and performance of the third resting EEG (duration: 2 m). The experimental protocol is shown in Figure 2 .

**Figure 3:**
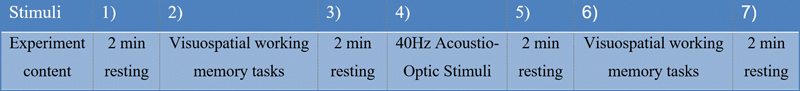
The experimental administration of acousto-optic stimuli.

The 40-Hz AOS experimental session was divided into 7 segments: performance of the first resting EEG (duration: 2 m); working memory task after no stimulation in the control experiment (duration: 8 m); performance of the second resting EEG (duration: 2 m); performance of the EEG with 40-Hz AOS (duration: 20 m); performance of the third resting EEG (duration: 2 m); working memory task after the 40-Hz AOS (duration: 8 m); and performance of the fourth resting EEG (duration: 2 m). The experimental protocol is shown in Figure 3 .

### Data analyses

Before the processing and analysis of EEG signals, it was important to perform procedures to eliminate interfering noise as follows:

- We identified the channel data;
- We eliminated extraneous data and excluded data segments with significant amplitude fluctuations;
- Filtering: A 50-Hz bandstop filter was used to eliminate power frequency interference, while a bandpass filter ranging from 0.1 Hz to 80 Hz was utilized to recover the desired signals within the target band interval from the EEG data;
- The reference electrode was repositioned to the average value of the left and right mastoids (M1, M2). It was important to note that varying reference sites might significantly influence the experimental outcomes;
- The removal of ocular artifacts and electrical noise was achieved by the application of techniques of independent component analysis (ICA), which is a blind source separation (BSS) algorithm that enables the separation of electrical noise components from the signal, hence facilitating the targeted removal of these specific noise components; and
- We removed the faulty segment.

The present study is on the impact of the operation process of working memory on the activation of the working memory system. To achieve this, we analyzed the task EEG data of the participants while they were engaged in a delayed matching-to-sample working memory task. We specifically examined the characteristics of the event-related potentials (ERPs) during the coding, retention, and decoding periods of working memory. The findings of the present study shed light on the different effects of 40-Hz stimulation on working memory processing across different periods of working memory.

Currently, the prevailing components of ERPs are P1, N1, P2, N170, N2, and P3, among others. These distinct components are regarded as indications of various working memory activities. The prevailing consensus is that P1 represents preattentive and automatic sensory reactions elicited by visual stimuli. 23 The sensitivity of N1 components to attention has been noted. 24 The P2 component of the brain's electrical activity has been associated with working memory and classification processes, and its fluctuations are linked to the specific tasks that are being performed. 25 The N170 component is widely recognized as a characteristic feature of face specificity. 26 The N2 component plays a role in the conflict monitoring process that occurs during cognitive control activities. 27

Several studies have indicated that one of the components of ERPs, known as P3, was linked to various cognitive functions, 28 which include attention resource allocation, working memory renewal, emotional processing, perceptual discrimination, decision-making, information processing, and conflict resolution. 29 These functions are considered indicators of processing ability. Furthermore, the amplitude of P3 has been found to be linked to various factors, including the probability of stimuli, the matching of stimuli, the renewal of working memory, and the readiness of response. 30 Numerous scholars hold the belief that the incubation period of P3 serves as a physiological measure of the time required for stimulation processing. 31 It has been observed that prolonging the duration of low-level sensory processing or high-level classification processing leads to an increase in the incubation period of P3. Conversely, a shorter incubation period results in a more rapid inhibition of external processes, potentially facilitating the transmission of information in working memory. 32 Given the clarity and reliability of the characteristics of the P3 component, as well as its well-established theoretical foundation, the present research aimed to investigate the mechanism through which 40-Hz stimulation affects working memory. We specifically used the amplitude and incubation period of the P3 component as indicators of the task-state EEG signal.

### Statistical analyses

The behavioral data obtained from various blocks were subjected to statistical analyses through paired t-tests to examine the impact of AOS on the behavioral performance of working memory.

To assess the impact of various stimuli on working memory, the right rate for each stimulus was computed for all participants. The statistical tests were conducted using repeated-measures analysis of variance (ANOVA) with a significance level of α = 0.05. The Mauchly test was conducted to assess the validity of the assumption of sphericity. Additionally, the Greenhouse-Geisser approximation was employed to adjust the degrees of freedom as needed.

## RESULTS

### ERPs

In contrast to spontaneous EEG, ERPs have considerably diminished amplitudes. The extraction of ERP components from the background noise of EEG involves the superimposition and averaging of numerous EEG waveforms elicited by the identical stimulus. Hence, in the present study, the collection of EEG data for further offline analysis involved the extraction of the initial 200 ms to the final 1,000 ms of the corresponding event marker. Subsequently, a superimposed average was computed.

Based on previous studies in the literature, a seven brain regions were identified as the focal points of investigation: the prefrontal lobe, encompassing the means of Fp1, Fp2, F7, and F8; the frontal lobe, represented by the means of F3, Fz, and F4; the central region, characterized by the means of C3, Cz, and C4; the temporal leaf, represented by the means of T7, T8, P7, and P8; the parietal lobe, encompassing the means of P3, Pz, and P4; the parieto-occipital lobe, represented by the means of PO3, POz, and PO4; and the occipital lobe, encompassing the means of O1 and O2. The group average plot of ERPs in various brain regions of working memory can be created by superimposing and averaging the ERPs of all individuals during the same working memory phase. In the present research, we used the method of superimposed averaging to obtain the ERP waveform associated with working memory.

The P3 components were divided into two groups according to their amplitude and latency: the stimulation group and the control stimulation group. The measurements were taken in the seven brain regions mentioned before. The repeated-measures ANOVA was followed by post-hoc testing to examine simple effects. In instances in which the data failed to satisfy the assumption of sphericity in the hypothesis testing, the Greenhouse-Geisser correction method was employed for adjustment. The findings were analyzed using the Bonferroni correction in the simple effects analysis, with a predetermined statistical significance level of *p* < 0.05.

### Statistical values of the P3 component for the coding period of working memory

The mean and standard deviation (SD) values for the amplitude of the P3 components of the acoustic stimuli and AOS obtained in various brain areas are presented in Table 1 .

**Table 1:** Mean P3 amplitudes in 7 brain regions during the coding period of acoustic and acoustic-optic stimuli

P3 amplitudes (μV)	40-Hz acoustic stimuli		Pink noise		40-Hz acoustic-optic stimuli		No stimuli	
	Mean	SD	Mean	SD	Mean	SD	Mean	SD
Prefrontal	3.007	± 8.191	1.426	± 6.613	2.396	± 8.102	0.727	± 6.371
Frontal	3.989	± 6.408	2.592	± 5.015	1.712	± 7.039	0.254	± 5.194
Central	6.233	± 5.678	5.459	± 4.347	4.329	± 6.491	2.61	± 5.416
Temporal	4.06	± 6.320	2.945	± 4.603	1.414	± 6.410	0.022	± 6.719
Parietal	9.277	± 4.599	7.993	± 3.863	6.325	± 5.317	4.922	± 5.447
Parieto-occipital	8.561	± 5.311	7.176	± 4.120	5.666	± 6.450	4.214	± 6.026
Occipital	5.249	± 3.717	4.852	± 3.767	2.621	± 5.435	2.162	± 5.673

Abbreviation: SD, standard deviation.

The repeated-measures ANOVA of the P3 peak amplitude of the acoustic Ssimuli showed that the main effect of group was significant: F (1, 19) = 31.735; *p* < 0.001; η _p_^2^ = 0.089. The amplitude of the P3 component in all brain areas during the presentation of acoustic stimuli was found to be considerably higher compared to the amplitude observed during the presentation of pink noise stimuli. The statistical values were: F _prefrontal_ (1, 19) = 12.016, p _prefrontal_ = 0.001; F _frontal_ (1, 19) = 6.973, p _frontal_ = 0.009; F _central_ (1, 19) = 4.504, p _central_ = 0.021; F _temporal_ (1, 19) = 6.403, p _temporal_ = 0.012; F _parietal_ (1, 19) = 4.004, p _parietal_ =0.046; F _parieto-occipital_ (1, 19) = 7.431, p _parieto-occipital_ = 0.007; and F _occipital_ (1, 19) = 4.082, p _occipital_ = 0.044. The P3 amplitude in the stimulation and control groups presented the greatest magnitude in the prefrontal lobe. Furthermore, the P3 amplitude in the prefrontal lobe showed the most substantial increase subsequent to the administration of acoustic stimuli.

The repeated-measures ANOVA of the P3 peak amplitude of AOS showed that the main effect of group was significant: F (1, 19) = 29.070; *p* < 0.001; ηp ^2^ = 0.070. The amplitude of the P3 component in all brain areas during the AOS was found to be considerably higher compared to the amplitude under non-Stimuli conditions. The statistical values were: F _prefrontal_ (1, 19) = 27.231, P _prefrontal_ < 0.001; F _frontal_ (1, 19) = 12.616, P _frontal_ = 0.035; F _central_ (1, 19) = 5.148, P _central_ = 0.024; F _temporal_ (1, 19) = 4.503, P _temporal_ = 0.034; F _parietal_ (1, 19) = 4.452, P _parietal_ = 0.038; F _parieto-occipital_ (1, 19) = 4.275, P _parieto-occipital_ = 0.043; and F _occipital_ (1, 19) = 3.932, P _occipital_ = 0.049. The stimulation and control groups presented the highest amplitude of P3 in the parietal lobe. Furthermore, the amplitude of P3 in the prefrontal lobe showed the greatest increase following the administration of AOS.

### Statistical values of the P3 component for the retention period of working memory

The mean and SD values for the amplitude of the P3 components of the acoustic stimuli and AOS obtained in various brain areas are presented in Table 2 .

**Table 2:** Mean P3 amplitudes in 7 brain regions during the retention period of acoustic and acoustic-optic s

P3 amplitudes (μV)	40-Hz acoustic stimuli		Pink noise		40-Hz acoustic-optic stimuli		No stimuli	
	Mean	SD	Mean	SD	Mean	SD	Mean	SD
Prefrontal	5.933	± 5.374	4.562	± 4.639	8.209	± 24.767	6.577	± 23.642
Frontal	3.221	± 4.524	2.055	± 3.962	7.658	± 15.836	6.251	± 13.157
Central	4.069	± 4.417	2.922	± 4.673	3.966	± 9.265	2.544	± 6.464
Temporal	1.126	± 2.972	0.060	± 4.181	1.82	± 8.372	0.646	± 7.321
Parietal	1.857	± 3.767	0.486	± 4.759	2.109	± 6.686	0.644	± 3.547
Parieto-occipital	1.319	± 3.750	0.201	± 4.652	1.645	± 5.384	0.914	± 3.333
Occipital	1.958	± 3.339	0.719	± 4.678	0.505	± 4.468	0.172	± 2.469

Abbreviation: SD, standard deviation.

The repeated-measures ANOVA of the P3 peak amplitude of acoustic stimuli showed that the main effect of group was significant: F (1, 19) = 29.099; *p* < 0.001; η _p_^2^ = 0.083. The amplitude of the P3 component in all brain areas during the presentation of acoustic stimuli was found to be considerably higher compared to the amplitude observed during the presentation of pink noise stimuli. The statistical values were: F _prefrontal_ (1, 19) = 17.331; p _prefrontal_ < 0.001; F _frontal_ (1, 19) = 3.856, p _frontal_ = 0.041; F _central_ (1, 19) = 16.215, p _central_ < 0.001; F _temporal_ (1, 19) =3.485, p _temporal_ =0.047; F _parietal_ (1, 19) = 6.182, p _parietal_ = 0.013; F _parieto-occipital_ (1, 19) = 5.427, p _parieto-occipital_ = 0.020; and F _occipital_ (1, 19) = 4.301, p _occipital_ =0.033. The P3 amplitude in the stimulation and control groups presented the greatest magnitude in the prefrontal lobe. Furthermore, the P3 amplitude in the prefrontal lobe showed the most substantial increase subsequent to the administration of acoustic stimuli.

The repeated-measures ANOVA of the P3 peak amplitude of the AOS showed that the main effect of group was significant: F (1, 19) = 7.299; *p* < 0.001; ηp ^2^ = 0.101. The amplitude of the P3 component in all brain areas during the AOS was found to be considerably higher compared to the amplitude under non-stimuli conditions. The statistical values were: F _prefrontal_ (1, 19) = 27.247, p _prefrontal_ < 0.001; F _frontal_ (1, 19) = 4.444, p _frontal_ =0.045; F _central_ (1, 19) = 5.190, p _central_ = 0.022; F _temporal_ (1, 19) = 4.961, p _temporal_ =0.032; F _parietal_ (1, 19) = 5.027, p _parietal_ = 0.026; F _parieto-occipital_ (1, 19) = 5.105, p _parieto-occipital_ = 0.024; and F _occipital_ (1, 19) = 4.569, p _occipital_ =0.042. The stimulation and control groups presented the highest amplitude of P3 in the parietal lobe. Furthermore, the amplitude of P3 in the prefrontal lobe presented the greatest increase following the administration of AOS.

### Statistical values of the P3 component results for the extraction period of working memory

The repeated-measures ANOVA of the P3 peak amplitude of acoustic stimuli showed that the main effect of group was not significant. (p > 0.05).

### Behavioral data

Mean and SD values were used to quantify the accuracy and reaction time across various stimuli. The statistical analysis of the stimulation and control groups involved the evaluation of the correct rate and reaction time using a paired *t* -test. The findings are presented in Table 3 . The mean accuracy of the stimulation group was found to be greater than that of the control group.

**Table 3:** Accuracy and reaction time under different stimuli

Stimuli	Accuracy			Reaction time		
	Mean	SD	*t* -test	Mean	SD	*t* -test
WS	0.684	0.128	*t* = 3.684	1278.969	895.228	*t* = -1.945
OS	0.807	0.095	*p* = 0.002*	1196.150	817.861	*p* = 0.052
PN	0.677	0.211	*t* = 2.843	1051.315	512.897	*t* = -0.713
AS	0.786	0.113	*p* = 0.011*	1032.506	439.874	*p* = 0.476
No stimuli	0.754	0.161	*t* = 3.285	1005.605	469.029	*t* = -1.756
AOS	0.857	0.079	*p* = 0.004*	958.893	490.147	*p* = 0.080

Abbreviations: AOS, acoustic-optic Stimuli; AS, acoustic stimuli; OS, optic stimuli; PN, pink noise; SD, standard deviation; WS, white-screen stimuli.

**Table 4:** Comparison of the accuracy and reaction time of different stimuli

Stimuli	*t* -test	
	Accuracy	Reaction time
AOS-AS	*t* = 2.392 *p* = 0.028*	*t* = -2.762 *p* = 0.006*
AOS-OS	*t* = 2.455 *p* = 0.024*	*t* = -6.232 *p* = 0.000*
AS-OS	*t* = -0.242 *p* = 0.812	*t* = -4.547 *p* = 0.000*

Abbreviations: AOS-AS, comparison between acoustic-optic stimuli and acoustic stimuli; AOS-OS, comparison between acoustic-optic stimuli and optic stimuli; AS-OS, comparison between acoustic stimuli and optic stimuli.

## DISCUSSION

Given the substantial body of previous research indicating that AS or AOS interventions have been found to improve working memory, cognitive functioning, attention, and other related factors, it was reasonable to anticipate notable disparities in behavioral performance and EEG features following exposure to AOS in comparison to alternative stimuli. The result was consistent with the anticipated outcome.

### ERPs

Following the stimulation, we observed an improvement in working memory capacity, resulting in a corresponding and directly proportional increase in P3 amplitude.

After the completion of an attentional-orienting exercise, the participants presented an improvement in the allocation of cognitive resources towards various tasks. Moreover, the allocation of attention specifically pertaining to the processing of target stimuli was heightened during the process of constructing representations. During the coding phase of working memory, we observed that the amplitude of the P3 component presented a greater increase after the administration of AOS. This finding suggests that the use of AOS facilitated the allocation of additional cognitive resources towards task performance. Furthermore, the improvement in attention allocation associated with target processing was evident during the encoding of representations. The retention period is the only phase of working memory in which external stimuli are absent, and the sole objective for the participants was to concentrate on the internal execution of the supplied items. The P3 component observed during this stage typically signifies the initiation of the memory maintenance process, evaluation of stimulation, and the ability to inhibit interference. As the stage progresses, a higher amplitude of P3 may indicate a greater inclination to suppress irrelevant interference and sustain attention to successfully accomplish the task of memory consolidation. Additional research has demonstrated that the allocation of attention resources by the central executive system of working memory is influenced by the overall level of arousal. The overall level of arousal plays a crucial role in determining the processing capacity available for attention allocation during ongoing tasks. Moreover, individuals with lower cognitive abilities exhibit a reduction in the amplitude of the P3 component. The present study examines the impact of auditory stimulation on the P3 amplitude during the encoding and retention periods. The findings suggest that there was a higher increase in P3 amplitude following auditory stimulation at 40 Hz, which may be attributed to the heightened level of arousal induced by the frequency. This heightened arousal level was believed to assist the attention allocation process, hence improving processing ability.

Research 33 34 on P3 components in different areas if the brain has consistently demonstrated that the parietal lobe is typically associated with P3 activation. Research on metabolism 33 has indicated that the parietal lobe frequently takes on a significant role in the allocation of attention throughout space and the cognitive processing of spatial relationships. Numerous studies 34 have provided empirical evidence supporting the involvement of the parietal cortex in encoding stimuli throughout a range of visual working memory tasks. This observation suggests that, regardless of the specific stimulus employed, the coding period consistently elicited the highest P3 amplitude inside the parietal lobe, as indicated by the findings of the present investigation.

### Behavioral data

A notable disparity in the precision of the outcomes of working memory tasks was observed between the stimulation and control groups. The accuracy after the administration of AOS was notably greater compared to what it was after the administration of acoustic or optic stimuli. A substantial reduction in the reaction time was also observed. This finding shows that the administration of 40-Hz AOS can significantly improve the working memory performance and memory capacity of the participants.

In conclusion, the possible mechanisms to improve working memory performance before and after 40-Hz AOS were analyzed from the perspective of the task-state EEG analysis. The results show a significant improvement in accuracy after the administration of 40-Hz AOS, which can be explained by the effectiveness of said stimulation to enhance the performance and cognitive ability of the working memory system.
